# Apolipoprotein E regulates mitochondrial function through the PGC-1α-sirtuin 3 pathway

**DOI:** 10.18632/aging.102516

**Published:** 2019-12-06

**Authors:** Junxiang Yin, Megan Nielsen, Tanner Carcione, Shiping Li, Jiong Shi

**Affiliations:** 1 China National Clinical Research Center for Neurological Diseases, Beijing Tiantan Hospital, Capital Medical University, Beijing, China; 2 Barrow Neurological Institute, St. Joseph Hospital and Medical Center, Dignity Health Organization, Phoenix, AZ 85013, USA; 3 School of Life Sciences, Arizona State University, Tempe, AZ 85281, USA; 4 Department of Neurology, The Second Hospital of Hebei Medical University, Shijiazhuang, China; 5 Advanced Innovation Center for Human Brain Protection, Capital Medical University, Beijing, China

**Keywords:** hypometabolism, sirtuin, apolipoprotein

## Abstract

Cerebral hypometabolism is a pathophysiological hallmark of Alzheimer’s disease (AD). Our previous studies found that a mitochondrial protein, sirtuin3 (Sirt3), was down-regulated in human AD postmortem brains. Sirt3 protected neurons against oligo-amyloid β-42 induced hypometabolism in human Apolipoprotein E4 (ApoE4) transgenic mice. However, how ApoE affects mitochondrial function and its proteins such as Sirt3 remains unclear.

We characterized and compared levels of Sirt3 and peroxisome proliferator-activated receptor gamma coactivator 1-alpha (PGC-1α, a Sirt3 activator), oxidative stress proteins, synaptic proteins, cognitive task performance and ATP production in 12-month old human ApoE4 and ApoE3 transgenic mice, and assessed changes in Sirt3 expression on cellular metabolism in primary neurons from ApoE4 and ApoE3 transgenic mice.

Compared to ApoE3 mice, Sirt3 and PGC-1α levels were significantly lower in ApoE4 mice. Learning and memory, synaptic proteins, the NAD+/ NADH ratios, and ATP production were significantly lower in ApoE4 mice as well. Sirt3 knockdown reduced the oxygen consumption and ATP production in primary neurons with the human ApoE3, while Sirt3 overexpression protected these damages in ApoE4 neurons.

Our findings suggest that ApoE4 suppresses mitochondrial function via the PGC-1α- Sirt3 pathway. This discovery provides us novel therapeutic targets for the treatment and prevention of AD.

## INTRODUCTION

Apolipoprotein E4 (ApoE4) is the major genetic risk factor for late-onset Alzheimer’s disease (AD) [1]. ApoE4 carriers have reduced glucose metabolism in brain and this reduction may develop at earliest stage of amyloid-β deposition [2–10]. We and others reported ApoE4 was associated with reduced ATP levels in the cerebral cortex and this reduction in cerebral energy production was detrimental to learning and memory in mouse models of AD [11–14]. In our previous studies, we found that a mitochondrial protein, sirtuin 3 (Sirt3), was down-regulated in human AD postmortem brains when compared to non-demented subjects [15, 16]. Increasing Sirt3 expression by genetic engineering improved energy production and neuroprotection against oligo-amyloid β-42 induced hypometabolism in ApoE4 transgenic mice [17, 18]. However, how ApoE affects mitochondrial function and Sirt3-related pathway remains unclear.

Peroxisome proliferator-activated receptor gamma coactivator-1 alpha (PGC-1α) is a transcriptional activator of Sirt3 expression [19, 20]. Exercise and fasting activate PGC-1α and Sirt3 and lead to enhanced mitochondrial function [21–23]. Thus, we hypothesized that ApoE4 down-regulates the PGC-1α-Sirt3 signal pathway and subsequently affects mitochondrial function.

To test this hypothesis, we characterized and compared protein levels of Sirt3 and PGC-1α, oxidative stress proteins, synaptic proteins, cognitive task performance and ATP production in human ApoE4 and ApoE3 transgenic mice, and assessed the effects of experimental modifications in Sirt3 expression on cellular metabolism in primary neurons from ApoE4 and ApoE3 transgenic mice.

## RESULTS

### ApoE regulates the PGC-1α-Sirt3 pathway

**Figure 1:**
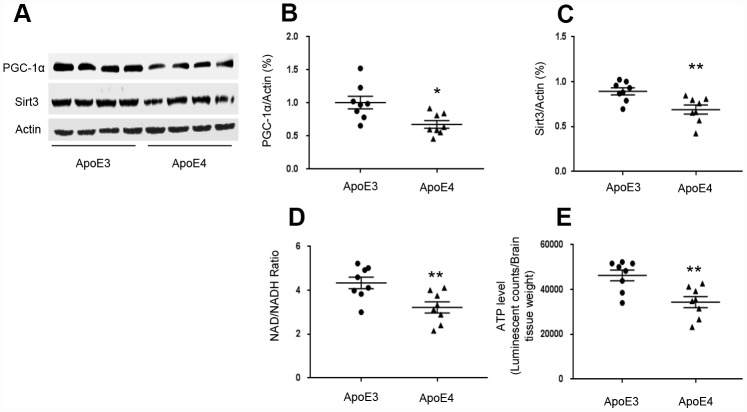
**ApoE regulates the PGC-1α-Sirt3 pathway.** Temporal lobes were freshly collected from 12-month-old ApoE4 mice and age-matched ApoE3 mice. (**A**–**C**) The levels of PGC-1^α^ and Sirt3 were evaluated and normalized with an internal control (β-actin) in Western blot. (**D**) NAD^+^/ NADH ratio was measured and analyzed using the NAD^+^/ NADH assay kit. (**E**) ATP levels were measured using the ATP assay kit and normalized with brain tissue wet weight (Luminescent counts/ brain tissue weight). N= 8 for each group, *p< 0.05, **p< 0.01.

We used transgenic mice that express homozygous human ApoE4 and age-matched human ApoE3 to test brain levels of Sirt3 and PGC-1^α^. We selected 12-month-old ε4 mice since they had shown cognitive impairment in previous studies [12]. In temporal lobes, PGC-1^α^ levels in ApoE4 mice (0.68± 0.06) were decreased compared to ApoE3 mice (1.01± 0.10, p= 0.010, Figure 1A and 1B). Sirt3 levels in ApoE4 mice (0.69± 0.05) were decreased compared to that in ApoE3 mice (0.89± 0.04, p= 0.007, Figure 1A and 1C). Since Sirt3 is an NAD^+^-dependent protein, which deacetylates and activates multiple substrates that are related with ATP production [24–26], we tested the effect of ApoE4 on NAD^+^/ NADH ratio and ATP production. The NAD^+^/ NADH ratio was reduced in ApoE4 mice (3.22± 0.26) compared with ApoE3 mice (4.34± 0.26, p= 0.009, Figure 1D). So were ATP levels in ApoE4 mice (34380± 2438), compared with age-matched ApoE 3 mice (46300± 2419, p= 0.004, Figure 1E).

### ApoE regulates oxygen consumption and ATP production via Sirt3

**Figure 2:**
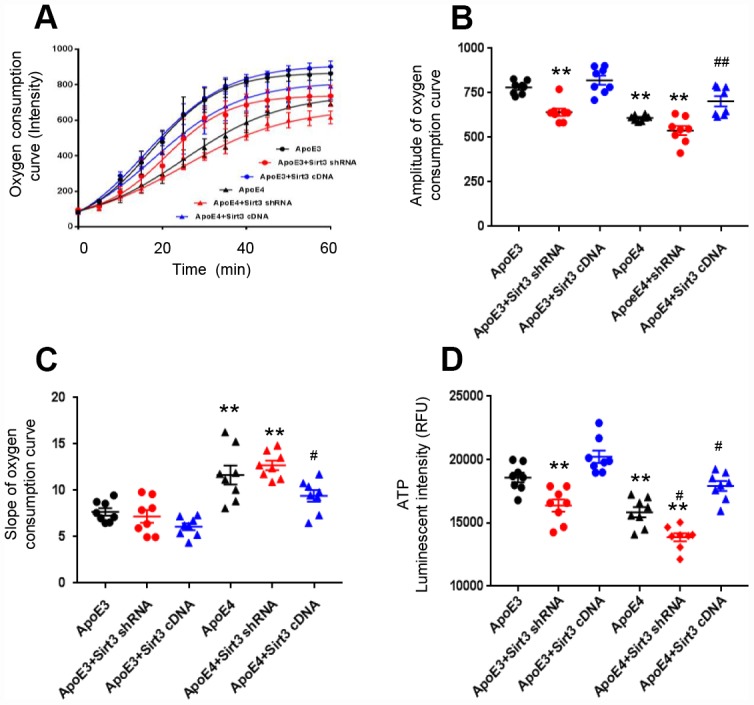
**ApoE regulates oxygen consumption and ATP production via Sirt3.** Primary cortical neurons from new born ApoE4 or ApoE 3 mouse brain were transfected by a lentivirus encoding Sirt3 shRNA (Sirt3 knockdown) or Sirt3 cDNA (Sirt3 overexpression). The oxygen consumption kinetics was analyzed. (**A**) Oxygen consumption curves; (**B**) Amplitude of oxygen consumption curves; (**C**) Slope of oxygen consumption curves. (**D**) ATP levels were measured in different groups (n= 8 in each groups, ** p< 0.01 compared to ApoE3 neurons, # p< 0.05 and ## p< 0.01 compared to ApoE4 neurons).

We cultured primary cortical neurons from newborn ApoE4 or ApoE3 mice and transfected them by lentivirus, encoding a Sirt3 shRNA (Sirt3 knockdown) or a Sirt3 cDNA (Sirt3 overexpression) sequence. Sirt3 is an important mitochondrial protein which regulates mitochondrial bioactivity. The oxygen consumption rate of the cell is a hallmark indicator of normal cellular function, and it is used as a parameter to study mitochondrial function. With Sirt3 overexpression or Sirt3 knockdown, we measured the oxygen consumption rate and ATP production of ApoE4 or ApoE3 neurons. The amplitude and slope of the curves of oxygen consumption kinetics were studied. The amplitude was defined as the difference between the max and the min in the curves of oxygen consumption kinetics. The amplitude represented the capacity of mitochondrial function, the bigger the amplitude, the stronger the capacity of mitochondrial function. The slope of oxygen consumption curves represented mitochondrial respiratory speed, the less the slope, the faster the mitochondrial respiratory speed. A higher amplitude and lower slope indicate a stronger cellular oxygen consumption rate. The oxygen consumption rate was reduced in ApoE4 neurons and ApoE3 neurons with Sirt3 knockdown; whereas it was improved in ApoE4 neurons with Sirt3 overexpression (Figure 2A). The group difference in the oxygen consumption rate was obvious in the amplitude (ApoE3: 780± 36.5, ApoE3+ Sirt3 shRNA: 641.4± 58.1, ApoE3+ Sirt3 cDNA: 820.7± 72.6, ApoE4: 609.3± 16.4, ApoE4+ Sirt3 shRNA: 538.3± 72.1, and ApoE4+ Sirt3 cDNA: 702.8± 79.7, Figure 2B); and in the slope (ApoE3: 7.69± 1.12, ApoE3+ Sirt3 shRNA: 7.20± 1.93, ApoE3+ Sirt3 cDNA: 6.08.± 1.03, ApoE4: 11.66± 2.87, ApoE4+ Sirt3 shRNA: 12.70± 1.43, and ApoE4+ Sirt3 cDNA: 9.41±1.75, Figure 2C). There were significant difference between ApoE3 and ApoE4 (p< 0.01), ApoE4 and ApoE4+ Sirt3 cDNA (p< 0.05) and to a less degree ApoE3 and ApoE3+ Sirt3 shRNA. Finally, when Sirt3 was knocked down in ApoE3 neurons, the ATP level was decreased (Figure 2D, p<0.01); and vice versa, when Sirt3 was overexpressed in ApoE4 neurons, the ATP level was increased (Figure 2D, p<0.01). These data provide strong evidence that ApoE regulates energy metabolism via Sirt3.

### ApoE regulates mitochondrial oxidative stress

**Figure 3:**
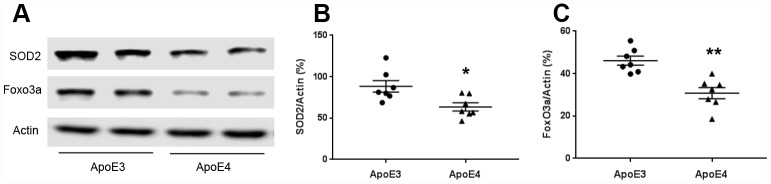
**ApoE regulates mitochondrial oxidative stress.** Brain tissues (temporal lobe) were collected from 12-month old ApoE3 and ApoE4 mice. Proteins involved in mitochondrial oxidative stress were measured and normalized with an internal control (β-actin) in Western blot. (**A**) Representative Western blots for SOD2 and Foxo3a were shown. (**B**) SOD2 and (**C**) Foxo3a protein levels were analyzed and plotted (n= 6-9 per group, * p< 0.05).

Mitochondria regulate reactive oxygen species production. We measured oxidative stress proteins SOD2 and Foxo3a. The protein levels of SOD2 were 88.4± 7.0 (ApoE3) and 63.5± 4.9 (ApoE4); and Foxo3a were 46.1± 2.1 (ApoE3) and 30.8± 2.7 (ApoE4) (Figure 3A–3C). SOD2 and Foxo3a were down-regulated in ApoE4 mice (p< 0.05).

### ApoE4 impairs synaptic integrity and cognitive function

**Figure 4:**
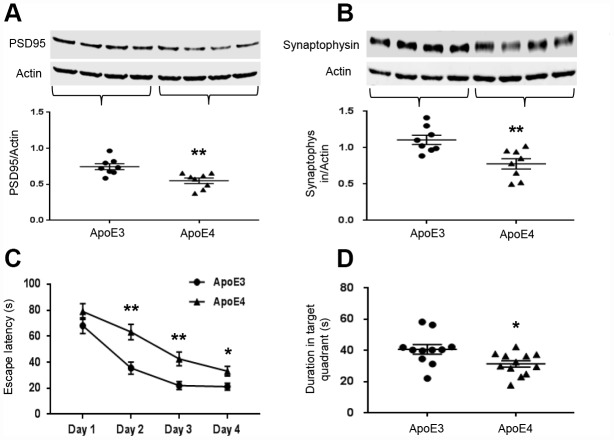
**ApoE4 impairs synaptic integrity and cognitive function.** Brain tissues (temporal lobe) were collected from 12-month old ApoE3 and ApoE4 mice. Synaptic proteins were measured and normalized with an internal control (β-actin) in Western blot. (**A**) Representative Western blot and plotted data of PSD95; (**B**) Representative Western blot and plotted data of Synaptophysin (n= 6-9 per group, **p< 0.01). The ability of learning and memory of ApoE4 mice and ApoE3 mice were also evaluated in MWM, the data of performance were analyzed for (**C**) the escape latency during 4-day learning period; (**D**) the time spent at the target quadrant on day 5 (n=11-12, * p< 0.05, **p< 0.01).

Since learning and memory are dependent on synaptic integrity, we measured the levels of synaptic-related proteins, PSD95 and synaptophysin. Both PSD95 (ApoE3: 0.75± 0.04 vs. ApoE4: 0.55± 0.04, p= 0.003, Figure 4A) and synaptophysin (ApoE3: 1.11± 0.06 vs. ApoE4: 0.78± 0.07, p=0.004, Figure 4B) were significantly reduced in ApoE4 mice. ApoE4 impaired the learning and memory ability of mice in the MWM task. The escape latency measures how fast the mouse can find the platform to escape out of the water maze. ApoE3 mice were faster than ApoE4 mice in learning after the second day of training (p< 0.01, Figure 4C), suggesting a faster learning curve. They also spent more time in the target quadrant even after the platform was removed, suggesting better delayed recall (40.8± 3.1s vs. 31.5± 2.1s, p= 0.018, Figure 4D).

## DISCUSSION

Our previous studies reported that mitochondria and its protein such as Sirt3 are important in regulating cerebral hypometabolism [17, 18]. In this study, we used human ApoE4 and ApoE3 targeted gene replacement mice, and genetically modified primary neurons from these transgenic mice to demonstrate that ApoE4 reduced ATP production by regulating the PGC-1α-Sirt3 signal pathway. This triggered mitochondrial oxidative stress and subsequently damaged synapses and caused cognitive impairment. This is the first report that identified a close link between ApoE4 and the PGC-1α-Sirt3 pathway.

PGC-1α expression was found to be decreased along with the clinical progression of dementia in the AD postmortem brain. This reduction in PGC-1α paralleled with an accumulation of amyloidogenic Aβ1-42 and Aβ1-40 peptides and phosphorylated tau proteins in cultured neurons derived from Tg2576 AD mice and in monkeys [27, 28]. Injection of PGC-1α in APP23 mice attenuated Aβ accumulation through reducing β-APP cleaving enzyme (BACE1) [29]. In human ApoE transgenic mice, genetic Ingenuity pathway analysis indicated that the PPAR-γ/ PGC-1α signaling pathway was activated in the ApoE2 brain and inhibited in the ApoE4 brain. In addition, PGC-1α overexpression ameliorated ApoE4-induced deficits in glycolysis and mitochondrial respiration [30]. PGC-1α activates Sirt3 expression [19, 20]. Because Sirt3 is NAD^+^-dependent, Sirt3 activity could be regulated by regulating the NAD^+^ level. ApoE4 expressing neurons had decreased the NAD+/NADH ratio, down regulated several electron transport chain (ETC) subunits, compromised mitochondrial function and reduced the ATP production [31]. Low ATP levels were found in the cortices of ApoE4 mice [13, 14]. ApoE4-induced reduction in NAD^+^ impedes Sirt3 to execute its deacetylase activity. Sirt3 knockout mice demonstrate hyperacetylated mitochondrial proteins and low levels of many ETC subunits [32]. On the other hand, activation of the Sirt3 energy pathway can improve total ATP production [18, 24–26]. These data demonstrate that ApoE regulates oxygen consumption and ATP production via the PGC-1α-Sirt3 pathway.

Mounting evidence indicates that ApoE4 may interfere with Aβ clearance [2, 3]. However, in aged ApoE4 transgenic mice, although cognitive abilities were impaired, no amyloid plaques were observed despite an increased total Aβ burden [11, 12]. Thus, other than Aβ, synaptic integrity likely plays an important role in preservation of memory and learning in this model. In this study, we have shown both PSD 95 and synaptophysin were down-regulated in ApoE4 mice. Sirt3 also mediated neuroprotective effects in neurons by regulating oxidative stress [33, 34]. We speculate Sirt3 exerts protective effects on synapses by reducing oxidative stress. Further studies are warranted to investigate the direct effect on Sirt3 on oxidative stress and synapses by using Sirt3 and ApoE double transgenic mice.

In conclusion, ApoE4 impairs mitochondrial biogenesis, causes oxidative stress and damages synapses to lead to cognitive deficits. Sirt3 overexpression improves mitochondrial function and ATP production in ApoE4 mice. Take together with our previous studies, these data provide further evidence that the PGC-1α-Sirt3 pathway plays a critical role in regulating ApoE4-induced cerebral hypometabolism and it may provide a novel strategy for the treatment and prevention of AD.

## MATERIALS AND METHODS

### ApoE transgenic mice

Transgenic mice carrying either human ApoE4 or ApoE3 were purchased from Taconic Biosciences, Inc. (Hudson, NY). They were generated as described previously [35, 36]. Briefly, mice deficient in ApoE (knockout, KO) were generated and maintained in the C57Bl/6 background. ApoE transgenic mice were generated by using microinjection of allele-specific human ApoE4 or ApoE3 genomic fragments to establish founders. The founders were then bred to ApoE KO mice lacking a functional mouse ApoE protein. These transgenic lines have been shown to transcribe and express appropriate human ApoE3 and ApoE4 protein in brain, liver, and other tissues without contamination of the endogenous mouse ApoE gene. We used 12-month-old homozygous mice for our experiments.

All mice were housed in a temperature and humidity-controlled vivarium, kept on a 12 hour dark/light cycle, and had free access to food and water. All experimental procedures were approved by the Institutional Animal Care and Use Committee of the Barrow Neurological Institute and performed according to the Revised Guide for the Care and Use of Laboratory Animals.

### Learning and memory tests

Spatial learning was assessed by the Morris water maze (MWM) task as described previously [37]. We labeled all mice with series number randomly. The person who performed these water maze tests was blinded to the number assignment. Briefly, each mouse was introduced into a circular pool and allowed to swim freely. The time (escape latency) required to reach the platform located in northeast quadrant, as well as the swimming speed was recorded in each trial. Once the mouse located the platform, it was permitted to stay on it for 10 seconds. If the mouse did not locate the platform within 120 seconds, it was placed on the platform for 10 seconds. The mouse was given four trials per day for 4 days with an inter-trial interval of 20 minutes. Each trial was initiated by randomly placing a mouse in one of the four starting locations. Escape latency and swimming speed were collected and analyzed using EthoVision® 3.1 tracking software (Noldus Information Technology Inc., Leesburg, VA). On the 5^th^ day, a single probe trial was carried out. In this trial, the platform was removed and each mouse was placed from southwest quadrant of the pool and allowed to swim for 120 seconds. The time spent in the target quadrant (northeast) was collected and calculated using EthoVision® 3.1 tracking software.

### Western blot

Fifty μg total proteins were used for western blotting. Primary antibodies were the following: anti-Sirt3 (#5490S, Cell Signaling Technology Inc., Danvers, MA), anti-peroxisome proliferator activated receptor gamma coactivator 1 alpha (PGC-1α, #NBP1-04676, Novus Biologicals Inc., Littleton, CO), anti-superoxide dismutase 2 (SOD2, Cell Signaling, Davers, MA), anti-forkhead box protein O3a (Foxo3a, Cell Signaling, Davers, MA), anti-postsynaptic density protein 95 (PSD-95, #3450, Cell Signaling Technology Inc.), anti-synaptophysin (#12270, Cell Signaling Technology Inc.), anti-β-actin (Santa Cruz, Dallas, TX), IRDye 800CW and IRDye 680CW antibodies (LI-COR Biosciences, Lincoln, NE). Immunoreactivity signals were quantified using Odyssey CLx. Protein levels were presented percentage relative to β-actin, an internal control.

### ATP measurement

Fresh mouse temporal brain tissue (20 mg) was collected and homogenized in 200 ul 2M perchloric acid on ice. Samples were kept on ice for 30 minutes. After the samples were centrifuged, the supernatant was collected and diluted the volume to 1000ul with ATP assay buffer. Then, the supernatant was transferred into two new 500ul tubes. Supernatant were neutralized to a pH between 7.0 and 7.6, and excess perchloric acid was precipitated with ice-cold potassium hydroxide (2M). After neutralization and centrifugation again, new supernatant were collected for ATP assay. For primary neurons growing on 96-well plates, they were treated with detergent and reconstituted substrate solution according to the manufacturer’s protocol. ATP levels were tested using a Luminescent ATP detection assay kit (#ab83355, Abcam, Cambridge, MA) according to the manufacturer’s protocol.

### NAD^+^ and NADH measurement

Fresh mouse temporal brain tissue (20 mg) was collected and homogenized in 400ul NAD^+^/ NADH extract buffer on ice. After the samples were centrifuged, the supernatant was collected into a new tube and deproteinized with perchloric acid (4M). After a second centrifuge and second supernatant transfer into a new tube, the supernatant were neutralized to a pH between 7.0 and 7.6 with ice-cold potassium hydroxide (2M). Then, samples were centrifuged and the supernatant were collected for total NAD^+^ and NADH assay. Total NAD^+^ and NADH were tested using an NAD^+^/ NADH assay kit (ab65348, Abcam) according to the manufacturer’s protocol. NAD^+^/ NADH ratio was calculated based on the value of total NAD^+^ and NADH (NAD^+^ = total NAD^+^ – NADH).

### Primary neuron culture

Primary cortical neurons were prepared from new-born pups of transgenic mice that carry either human ApoE4 or ApoE3. Cortical neurons were plated on poly-D- lysine coated glass coverslips in Neurobasal-media supplemented with 0.5% (w/v) L-glutamine, 1% Penicillin-Streptomycin 5% fetal bovine serum and 2% B27 supplement (Invitrogen) and medium was partially replaced every 4 days [37]. Primary neurons were transfected with lentivirus with Sirt3 cDNA or shRNA on day 7 [15]. On day 14, oxygen consumption and ATP levels were evaluated with MitoXpress Xtra-oxygen consumption assay and Luminescent ATP Detection Assay Kit.

### Vector construction and transfection

Over-expression or knock down of Sirt3 was performed as previously described [38]. Briefly, we subcloned exogenous mouse Sirt3 cDNA sequence into Lenti-CMV-GFP vector to over-express Sirt3. A short sequence consisting of 19 nucleotides targeting Sirt3 location 764 was constructed into an OmicsLink RNA expression clone in order to effectively knock down the expression of Sirt3. The shRNA vector, Lenti-Sirt3 vector, and a control vector were packaged into a third generation Lenti-Virus transfection system. After the cortical neurons matured in a petri dish for 7 days, we added transfecting viral particles at a multiplicity 100 infection. All vectors contain sequence of eGFP so the effectiveness of transfection can be visualized after 2-5 days. The levels of knocking down and over-expression were confirmed by Western blot [15].

### Oxygen consumption test in primary neurons

Oxygen consumption of primary cultured neurons was tested on day 14 using MitoXpress Xtra-oxygen consumption assay (MX-200, Luxcel biosciences, Ireland) as previously described [37, 38]. The probe fluorescence intensity of every well on 96 wells plate was recorded every 5 minutes for total 60 minutes on TECAN Spectra Fluor Detector (Tecan Group Ltd. Switzerland). The fluorescence signal intensity correlated with neurons’ oxygen consumption of wells. For 96 wells plate, fluorescence intensity based on every well (n=6-8 wells per group) from 0 minutes to 60 minutes were collected. Then, all data was analyzed using a Nonlinear regression (Curve fit)-Boltzmann sigmoidal on GraphPad Prism 7.03 software. Through Boltzmann sigmoidal analysis, the oxygen consumption curve (intensity) was produced and the value (the slope, maximal fluorescence intensity and minimal fluorescence intensity) were calculated for every well and group. The amplitude was recalculated through maximal fluorescence intensity minus minimal fluorescence intensity and represented the capacity of mitochondrial function. The slope represented mitochondrial respiratory speed.

### Statistical analysis

We applied unpaired T-test to analyze data from two groups and one way ANOVA with Tukey’s multiple comparison tests to compare values across multiple groups using GraphPad Prism version 7.03. All data were expressed as the mean ± SEM. Statistical significance was set at p< 0.05.
